# Publisher Correction: An expression atlas of variant ionotropic glutamate receptors identifies a molecular basis of carbonation sensing

**DOI:** 10.1038/s41467-020-14882-0

**Published:** 2020-02-25

**Authors:** Juan Antonio Sánchez-Alcañiz, Ana Florencia Silbering, Vincent Croset, Giovanna Zappia, Anantha Krishna Sivasubramaniam, Liliane Abuin, Saumya Yashmohini Sahai, Daniel Münch, Kathrin Steck, Thomas O. Auer, Steeve Cruchet, G. Larisa Neagu-Maier, Simon G. Sprecher, Carlos Ribeiro, Nilay Yapici, Richard Benton

**Affiliations:** 1 Center for Integrative Genomics, Faculty of Biology and Medicine, University of Lausanne, Génopode Building, Lausanne, CH-1015 Switzerland; 2 Department of Neurobiology and Behavior, Cornell University, W153 Mudd Hall, Ithaca, NY 14853 USA; 3 Champalimaud Centre for the Unknown, Lisbon, 1400-038 Portugal; 4 Department of Biology, Institute of Zoology, University of Fribourg, Chemin du Musée 10, Fribourg, CH-1700 Switzerland; 5 Present Address: Centre for Neural Circuits and Behaviour, University of Oxford, Tinsley Building, Mansfield Road, Oxford, OX1 3SR United Kingdom

**Keywords:** Genetics, Neuroscience

Correction to: *Nature Communications* 10.1038/s41467-018-06453-1, published online 12 October 2018.

The original version of this Article incorrectly omitted the received/accepted dates of Received: 08 March 2018; Accepted: 06 September 2018. This has now been corrected in the PDF and HTML versions of the Article.

