# Supplementary material for: An expression atlas of variant ionotropic glutamate receptors identifies a molecular basis of carbonation sensing
Source: Nat Commun. 2018 Oct 12;9:4252. doi: 10.1038/s41467-018-06453-1 (PMC6185939; doi:10.1038/s41467-018-06453-1)
Supplement: Supplementary file 1 — Supplementary Information [file 41467_2018_6453_MOESM1_ESM.pdf]

Supplementary Information

An expression atlas of variant ionotropic glutamate receptors identifies a molecular basis of carbonation sensing

Juan Antonio Sánchez-Alcañiz, Ana Florencia Silbering, Vincent Croset, Giovanna Zappia, Anantha Krishna Sivasubramaniam, Liliane Abuin, Saumya Yashmohini Sahai, Daniel Münch, Kathrin Steck, Thomas O. Auer, Steeve Cruchet, G. Larisa Neagu-Maier, Simon G. Sprecher, Carlos Ribeiro, Nilay Yapici and Richard Benton

Supplementary Table 1. Oligonucleotide sequences and construction of *Ir-Gal4* lines.

Gene[1]	Forward primer (5'-3')[2]	Reverse primer (5'-3')[2]	Length (bp)	Template	Vector	Integration site	Transgenic line code	Source[3]
Ir7a	AGATCTGGTGAAGAATAGAGTGTGGC	GAATTCTTTGAAACGAAACTGTTGCG	2318	OR genomic DNA	*pGAL4 attB*	*attP2*	BT58.1	(a)
Ir7b	AGATCTGGGATGAGAACGACATCGAT	GAATTCGGCTAAAGAGTTGCCAAAGG	578	OR genomic DNA	*pGAL4 attB*	*attP2*	BT47.1	(b)
Ir7c	CTCGAGGCCGGTTAGTGGTCCAAATA	AGATCTATCGTGTTTCATCGGTGGCT	1595	OR genomic DNA	*pGAL4 attB*	*attP2*	BT110.1	
Ir7d	AGATCTAACTTGTGTCAATGCGATCC	AGATCTGGCGAATGTGAAACATTTGG	1010	OR genomic DNA	*pGAL4 attB*	*attP2*	BT79.1	(b)
Ir7e	AGATCTTACTTCGGCAGAGGAACTAG	GAATTCTTGCTCCCCGGACAAATCGT	600	OR genomic DNA	*pGAL4 attB*	*attP2*	BT59.1	(b)
Ir7f	AGATCTGTCCGTCTATCGAAATCCGG	AGATCTATCGATCCTCGAATTCTCCA	766	OR genomic DNA	*pGAL4 attB*	*attP2*	BT98.1	
Ir7g	AGATCTATCGATCCTCGAATTCTCCA	AGATCTGTCCGTCTATCGAAATCCGG	766	OR genomic DNA	*pGAL4 attB*	*attP2*	BT99.1	(b)
Ir10a	GCGGCCGCGACACTATAGGTCCACTACC	GCGGCCGCTCGTATGGGATTTGTTAGCAC	2429	OR genomic DNA	*pGAL4 attB*	*attP2*	BT96.1	
Ir11a	AGATCTATGTATGTCATGCCACCAGC	GAATTCGACTGAATGGCCGTTGTGAA	2099	OR genomic DNA	*pGAL4 attB*	*attP2*	BT51.1	(a)
Ir20a	AGATCTACATTGTTCGGCACTCGCAG	AGATCTGTCGCCGGCATCGAAGGAAT	2488	OR genomic DNA	*pGAL4 attB*	*attP2*	BT61.1	
Ir47a	AGATCTGCTGAGTTGGGTGACGAATC	GAATTCTTTTTATGGCCTTTTGAAAC	2488	OR genomic DNA	*pGAL4 attB*	*attP2*	BT53.1	
Ir47bΨ								
Ir48aΨ								
Ir48b	AGATCTCCAGTCCAGTTCCAGATTGC	AGATCTCTGAAAGATATATAGAGCGT	2575	OR genomic DNA	*pGAL4 attB*	*attP2*	BT97.1	
Ir51aΨ								
Ir51b	AGATCTCAACCAATCAAGCTGGATAC	AGATCTGGTGGTTGATTCAATTGTGACA	2499	OR genomic DNA	*pGAL4 attB*	*attP2*	BT82.1	
Ir52a	AGATCTCCGACATTTCTTCGCGTAAC	AGATCTCACGAAACTGTTGACAATCC	2931	OR genomic DNA	*pGAL4 attB*	*attP2*	BT100.1	
Ir52b	AGATCTACTGGAGATATTGCGTTGCG	GAATTCTGTTTTCAAACAAACTGTTT	473	OR genomic DNA	*pGAL4 attB*	*attP2*	BT55.1	(a)
Ir52c	AGATCTAAACGCTGGATGAAATTCCG	AGATCTGGTGCTAAAGTGACTAATGG	644	OR genomic DNA	*pGAL4 attB*	*attP2*	BT80.1	
Ir52d	AGATCTTGAGATACTGGAGGAACTGC	GAATTCGGTGCAAGAGTTACTATTGC	664	OR genomic DNA	*pGAL4 attB*	*attP2*	BT56.1	
Ir52e[4]								
Ir54a	AGATCTGACGCAAGTCGACTCATTTG	AGATCTGTCCTTTCAATTATGTTGCC	2493	OR genomic DNA	*pGAL4 attB*	*attP2*	BT101.1	
Ir56a[5]	AGATCTATCATCACTGGCTGTCATGC	GAATTCGGCTGCCTTACCACTTTGAC	2400	OR genomic DNA	*pGAL4 attB*	*attP2*	BT52.1	(a)
Ir56b	GCGGCCGCATATCCTTCGGTCGAAGTGC	GCGGCCGCTGAAATAATTCTGCACTTGA	2461	OR genomic DNA	*pGAL4 attB*	*attP2*	BT62.1	
Ir56c	AGATCTGCAAAGACGTCCACAGTATG	GAATTCGACTTTCCCTTAGAAGCACC	319	OR genomic DNA	*pGAL4 attB*	*attP2*	BT57.1	(b)
Ir56d[6]	GAATTCATGAGCAGTGCAACATGCTC	AGATCTATATTTGTACGGCGACTGCC	858	OR genomic DNA	*pGAL4 attB*	*attP2*	BT76.1	
Ir56eΨ								
Ir60a[7]	GAATTCTAGTCCGCGGACTGATTATC	GAATTCTATTGCTTCTGTCACGTCGG	2523	OR genomic DNA	*pGAL4 attB*	*attP2*	BT83.1	
Ir60b	AGATCTCATACGATTTCCCGAACAGC	AGATCTTTTCGAGTTGTCTGCTCTGG	2368	OR genomic DNA	*pGAL4 attB*	*attP2*	BT104.1	
Ir60cΨ	GAATTCGATTGGATACCACAGGTGGC	GAATTCGGCGACTATCCGAAACGAGC	560	OR genomic DNA	*pGAL4 attB*	*attP2*	BT81.1	(b)
Ir60d	AGATCTAGATTGGGTACCACAGATGG	GAATTCTTTTAAGGCGACTGCTCACA	339	OR genomic DNA	*pGAL4 attB*	*attP2*	BT63.1	
Ir60e	AGATCTAAATAATGAGCAGTCCCGAT	GAATTCAAGGCAGCGGGAAAATGCTT	2466	OR genomic DNA	*pGAL4 attB*	*attP2*	BT72.1	
Ir60fΨ								
Ir62a	GAATTCAAATCACCGAGTTCAATGGC	GAATTCATTTTCGCTCGTGAACCATG	2439	OR genomic DNA	*pGAL4 attB*	*attP2*	BT118.1	
Ir67a	AGATCTACAGACGTTTATCAGCAAAG	GAATTCATATCCTGGCTGAATGGCTG	2496	OR genomic DNA	*pGAL4 attB*	*attP2*	BT73.1	
Ir67b	AGATCTTGGTGTGTCAGCACTATAGC	GAATTCTGAAATGTCTCTGAAATCCT	515	OR genomic DNA	*pGAL4 attB*	*attP2*	BT74.1	
Ir67c	GCGGCCGCGGTGCTCCATCGTATCCTTC	GCGGCCGCGATGCAGTCCTGCCCGAAAA	2736	OR genomic DNA	*pGAL4 attB*	*attP2*	BT84.1	
Ir68b	AGATCTCCGGTTACTCGAAAGATATG	GAATTCGTTCTACGAGCAGACTAACC	637	OR genomic DNA	*pGAL4 attB*	*attP2*	BT48.1	
Ir85a	AGATCTAAGTCCTTCCTCAGTTGTCC	GAATTCTGCAATGCCAACTGTTTGAG	1369	OR genomic DNA	*pGAL4 attB*	*attP2*	BT49.1	
Ir87a	CTCGAGAGTTACCCATATGGACACCG	AGATCTGCCGCAAACGAATGACTGAT	2097	OR genomic DNA	*pGAL4 attB*	*attP2*	BT122.1	
Ir94a	AGATCTGCACACAGATAGATTCGCAG	AGATCTTTTCTACTTTAGCCAACAAT	2294	OR genomic DNA	*pGAL4 attB*	*attP2*	BT64.1	
Ir94b	AGATCTAAGATCAAGCGAAGATGACG	GAATTCATTTCGTAATTCACGTAGTG	482	OR genomic DNA	*pGAL4 attB*	*attP2*	BT77.1	
Ir94c	AGATCTTTCTGGCGAGCGTCTCTATC	AGATCTTTTTAGTTAGCCTTGGGTTA	2303	OR genomic DNA	*pGAL4 attB*	*attP2*	BT65.1	
Ir94d	CTCGAGACATTGTGTTCGGGTACGTG	AGATCTTTTGAATGTGGGAATAGTTGGT	2420	OR genomic DNA	*pGAL4 attB*	*attP2*	BT111.1	
Ir94e	AGATCTTTGGCGACATAAGATGTGGC	GAATTCTTCCCAGGGGATTACACAAA	322	OR genomic DNA	*pGAL4 attB*	*attP2*	BT78.1	(b)
Ir94f	AGATCTGATTGTGGAGCGATCGATTG	GAATTCCTGTGCAGACGATGATGATG	2493	OR genomic DNA	*pGAL4 attB*	*attP2*	BT102.1	
Ir94g	GAATTCGAGCTCACTGTTCACTATGC	GAATTCCTTATAATACTGACTTCATT	388	OR genomic DNA	*pGAL4 attB*	*attP2*	BT75.1	
Ir94h	GAATTCTTGTTCACGCGGCAATTACG	GAATTCGACTTATACCGAAACCGACG	2000	OR genomic DNA	*pGAL4 attB*	*attP2*	BT60.1	(b)
Ir100a	AGATCTTTCATCGGAGTCGTAGCTAG	GAATTCGTCAGGAGTTACTGAACCGT	512	OR genomic DNA	*pGAL4 attB*	*attP2*	BT50.1	(a)

Footnotes

1: Ψ = predicted pseudogene in the reference *D. melanogaster* genome (http://flybase.org/); for most of these we therefore did not construct a driver line. For *Ir48a-Gal4* we observed very variable expression in the central and peripheral nervous system. For *Ir60c*, this locus is predicted to be intact in a w^{1118} strain.

2: Restriction enzyme sites in cloning primers are highlighted in blue.

3: Lines previously published: (a) Croset *et al*., PLOS Genetics (2010) (Ref. 15); (b) Croset *et al*., Scientific Reports (2016) (Ref. 28).

4: *Ir52e* is not present in the reference *D. melanogaster* genome, so no driver line was constructed for the locus in this study (see also Koh *et al*., Neuron (2014) (Ref. 26)).

5: *Ir56a-Gal4* displays expression in several olfactory sensory neuron populations; this is likely to be non-specific as there is no evidence for antennal expression of *Ir56a* (http://flybase.org/); as *Ir56a* is located within the intron of another gene (*5-HT1A*), this may reflect overlapping regulatory elements of these genes.

6: *Ir56d-Gal4* was also detected in some larval head chemosensory neurons, but this expression is weak and was not confirmed in the $Ir56d^{Gal4}$ reporter allele.

7: *Ir60a-Gal4* displays extensive and variable non-neuronal expression, which is likely to be non-specific; as *Ir60a* is located within the intron of another gene (*nord*), this may reflect overlapping regulatory elements of these genes.

Supplementary Table 2. Gustatory stimuli.

		Calcium imaging Figure 5c	
**Tastant**	**CAS**	**Concentration**	**Notes**
water	-	-	
glycerol	56-81-5	10% (v/v)	
fructose	57-48-7	1 M	
trehalose	6138-23-4	1 M	
sucrose	57-50-1	1 M	
caffeine	58-08-2	15 mg/ml	
denatonium	3734-33-6	10 mM	
arginine	74-79-3	100 mM	
histidine	71-00-1	100 mM	
lysine	56-87-1	100 mM	
aspartic acid	56-84-8	100 mM	
glutamic acid	56-86-0	100 mM	
acetic acid	64-19-7	1% (v/v)	
NaCl (high)	7647-14-5	1 M	
NaCl (low)	7647-14-5	10 mM	
$NaHCO_3$ (pH 5)	144-55-8	200 mM	0.5 ml of 200 mM $NaHCO_3$ (pH 6.5) + 50 μl of 5 M H_2PO_4
$CsHCO_3$ (pH 7)	29703-01-3	200 mM	pH set with HCl immediately before use
NaH_2PO_4	13472-35-0	500 mM	
PBS pH 4	-	100%	7.8 mM NaH_2PO_4 + 12.2 mM Na_2HPO_4 + 153.8 mM NaCl (pH set with HCl or NaOH)
PBS pH 7	-	100%	
PBS pH 10	-	100%	
carbonated water	-	-	Aproz ® (in mg/ml: Ca^{2+} 360; Mg^{2+} 70; Na^+ 6; K^+ 2.5; HCO_3^- 250; NO_3^- 1.5; SO_4^{2-} 930; $Si0_2$ 7) + gaseous CO_2
non-carbonated water	-	-	Aproz ® (in mg/ml: Ca^{2+} 360; Mg^{2+} 70; Na^+ 6; K^+ 2.5; HCO_3^- 250; NO_3^- 1.5; SO_4^{2-} 930; $Si0_2$ 7)
hexanoic acid	142-62-1	-	

Supplementary Table 3. Oligonucleotide sequences used for CRISPR/Cas9-mediated editing of the *Ir56d* locus.

Oligonucleotide	Sequence (5'-3')	Notes
CRISPRsgR	AAAAGCACCGACTCGGTGCCACTTTTTCAAGTTGATAACGGACTAGCCTTATTTTAACTTGCTATTTCTAGCTCTAAAAC	
CRISPRsgF-*Ir56d*[1]	GAAAT*TAATACGACTCACTATA*GGTCATCACGGAGCGCATGTGTTTTAGAGCTAGAAATAGC	T7 promoter sequence is italicised; *Ir56d* target sequence is underlined
CRISPRsgF-*Ir56d*[2]	GAAAT*TAATACGACTCACTATA*GGTCAGCTATAGCTATCCCATGTTTTAGAGCTAGAAATAGC	T7 promoter sequence is italicised; *Ir56d* target sequence is underlined
*Ir56d*sgRNAs-fwd	GCGGCCCGGGTTCGATTCCCGGCCGATGCAAAATATGGATAATCGTGCTGGTTTTAGAGCTAGAAATAGCAAG	sgRNA cloning into *pCFD5*
*Ir56d*sgRNAs-rev	ATTTTAACTTGCTATTTCTAGCTCTAAAACGCAAGCCCAGATCGTTTCTCTGCACCAGCCGGGAATCGAACCC	sgRNA cloning into *pCFD5*
*Ir56d*Gal4-HA1-fwd	GATCCACCTGCGATCTCGCCCCACGCACTGTGCATCCTTGAAGTGC	Homology Arm 1-*Gal4* ORF fusion
*Ir56d*Gal4-HA1-rev	GATCCACCTGCGATCCTACGGATCCAGATCCACTAGTCAAGGCAC	Homology Arm 1-*Gal4* ORF fusion
*Ir56d*Gal4-HA1-internal-fwd	ACTGGCAGTCGCCGTACAAATATGAAGCTACTGTCTTCTATCGAACAAGC	Homology Arm 1-*Gal4* ORF fusion
*Ir56d*Gal4-HA1-internal-rev	CGATAGAAGACAGTAGCTTCATATTTGTACGGCGACTGCCAGTGGGTAAC	Homology Arm 1-*Gal4* ORF fusion
Ir56d-HA2-fwd	GATCGCTCTTCGTATAGCCCAGATCGTTTCTCAGGCGCTTCATG	Homology Arm 2
Ir56d-HA2-rev	GATCGCTCTTCGGACGATGCCTTGCAATTGATACGTGAACG	Homology Arm 2

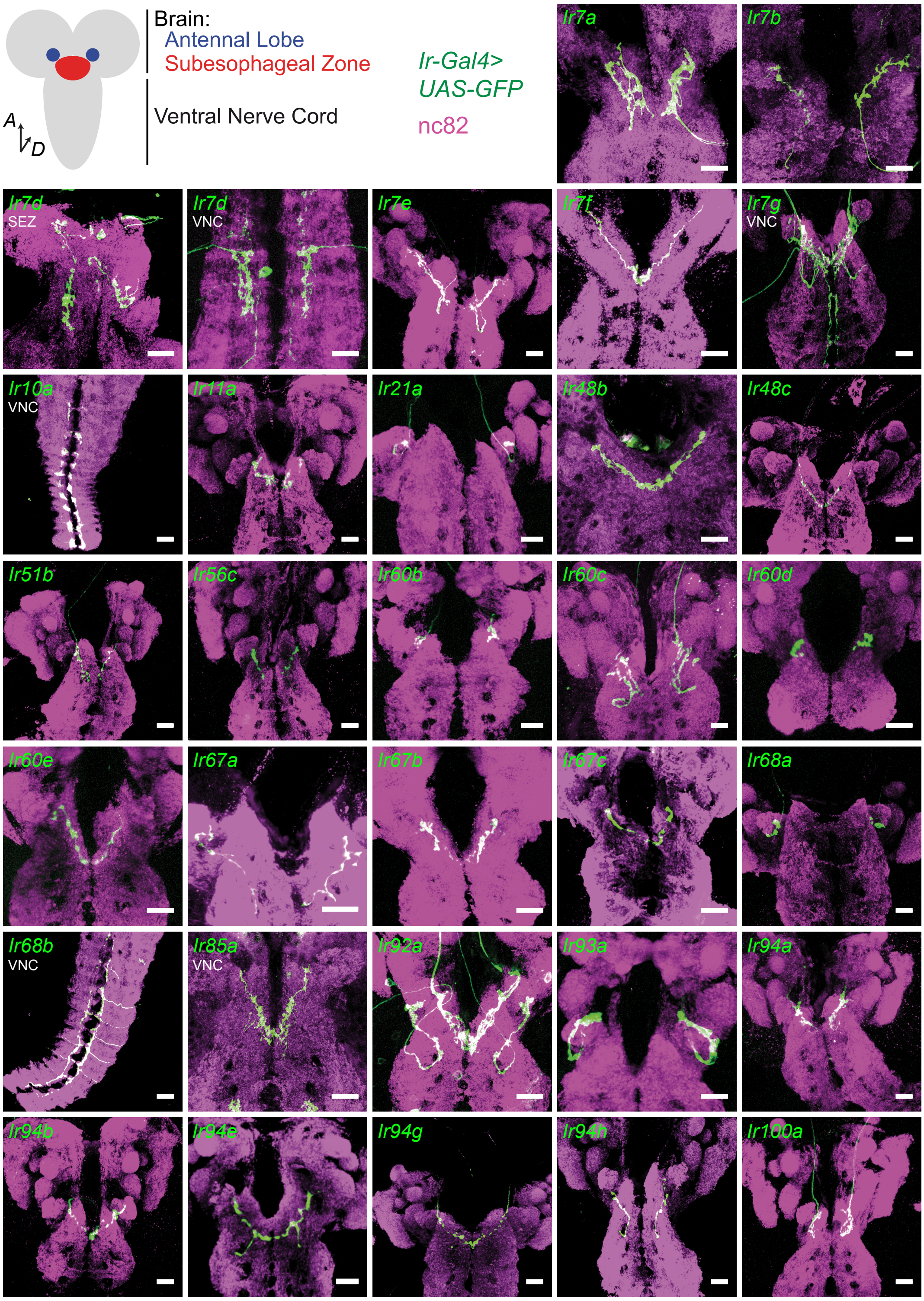

Supplementary Figure 1. Projections of *Ir-Gal4* expressing sensory neurons in the larval central nervous system.
Immunofluorescence with anti-GFP (green) and nc82 (magenta) on whole-mount brains of third instar larvae, revealing the projection patterns of *Ir-Gal4*-expressing neuron populations in the brain and ventral nerve cord (as schematised in the cartoon at the top left). Images for *Ir7a*, *Ir7b*, *Ir7g*, *Ir94e* and *Ir94h* drivers have been adapted from Ref. 28. Genotypes are of the form: *w;UAS-mCD8:GFP;IrX-Gal4*. SEZ: Subesophageal Zone; VNC: Ventral Nerve Cord. Scale bars: 20 μm.

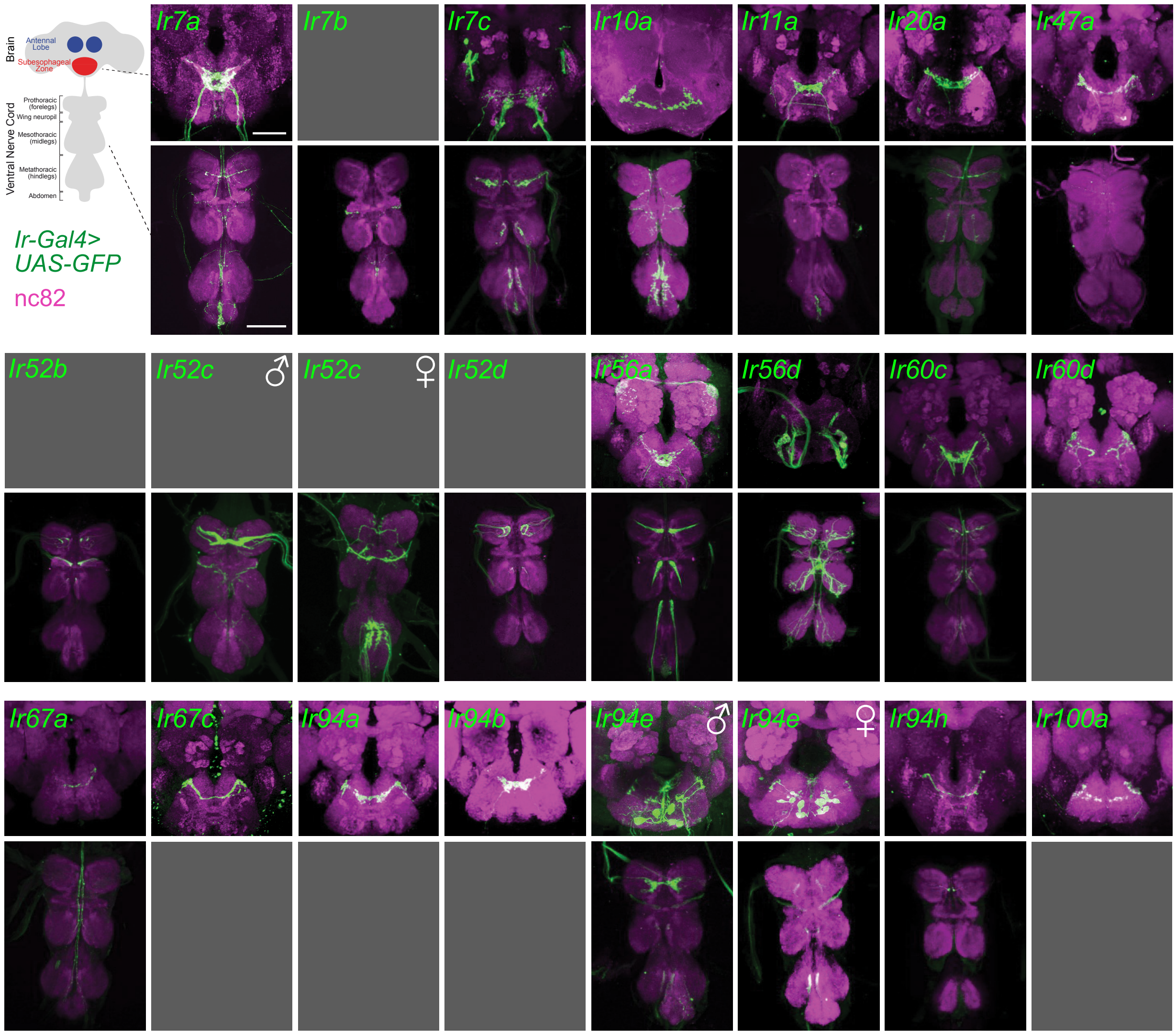

Supplementary Figure 2. Projections of *Ir-Gal4* expressing sensory neurons in the adult central nervous system.
Immunofluorescence with anti-GFP (green) and nc82 (magenta) on whole-mount adult brains and ventral nerve cords (schematised top left), revealing the projection patterns of *Ir-Gal4*-expressing neuron populations. Grey panels indicate no expression was detected for that Gal4 driver. Genotypes are of the form: *w;UAS-mCD8:GFP;IrX-Gal4*. Scale bars: 50 μm (brain), 100 μm (ventral nerve cord).

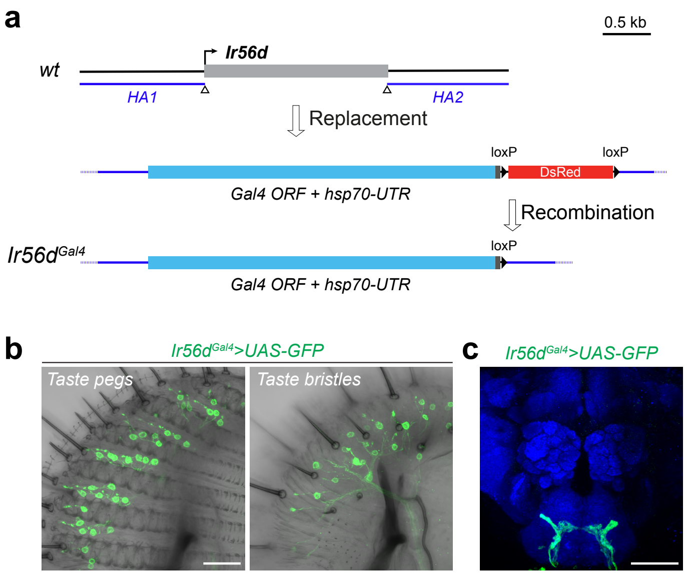

Supplementary Figure 3. Construction and characterisation of an *Ir56d*^{Gal4} allele.
(a) Schematic representing the generation of the *Ir56d*^{Gal4} allele. The entire *Ir56d* exon was substituted with the *Gal4* sequence by CRISPR/Cas9-mediated homologous recombination (HA: Homology Arm; open arrowheads indicate the positions of the sgRNA target sequences). Subsequently, the DsRed marker used for screening of positive recombination events was removed with Cre recombinase.
(b) Immunofluorescence with anti-GFP (green), overlaid on a bright-field image, of a whole mount proboscis of a *w;Ir56d*^{Gal4}*;UAS-mCD8:GFP* animal. Scale bar: 25 μm.
(c) Immunofluorescence with anti-GFP (green) and nc82 (blue) on a whole mount brain of a *w;Ir56d*^{Gal4}*;UAS-mCD8:GFP* animal. Scale bar: 50 μm.

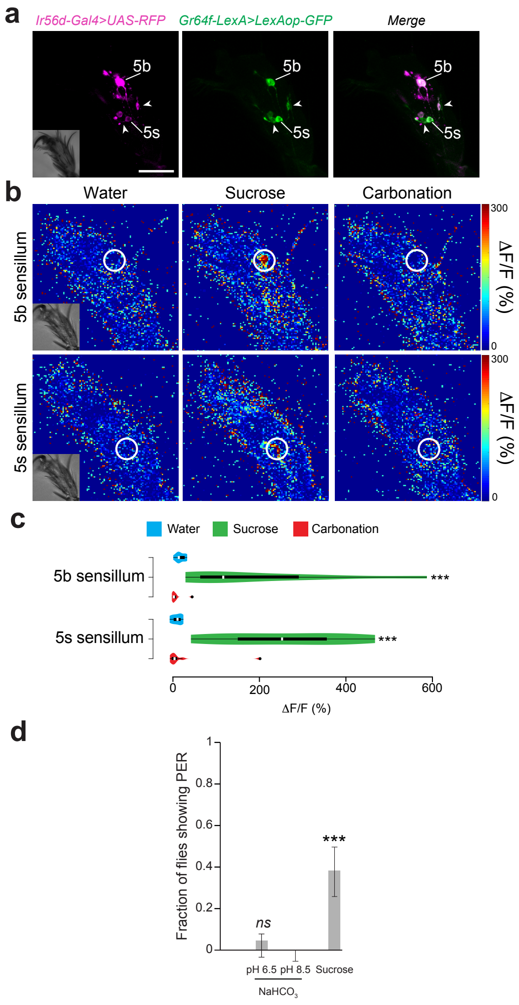

Supplementary Figure 4. IR56d neurons in the legs do not respond to carbonation.

(a) Raw fluorescence images of a whole-mount leg of a *w;LexAop-mCD8:GFP-2A-mCD8:GFP/UAS-mCD8:RFP;Gr64f-LexA/Ir56d-Gal4* animal. *Ir56d-Gal4* is expressed in a subset of *Gr64f-LexA* neurons; arrowheads indicate neurons that express *Gr64f-LexA* but not *Ir56d-Gal4*. The inset in the left-hand panel shows a bright-field view of the imaged tissue. Scale bar: 25 μm.

(b) Colour-coded images of ΔF/F (reflecting the maximal GCaMP3 fluorescence intensity changes; scale bars on the far right) of the responses of IR56d neurons in two different tarsal sensilla upon application of distilled water, 100 mM sucrose and a carbonated solution to the legs. White circles highlight the ROIs used for quantification of responses. The insets in the left-hand panels show bright-field views of the imaged tissue. Genotype: *w;UAS-GCaMP3;Ir56d-Gal4*.

(c) Quantification of changes in ΔF/F in the ROIs shown in (b) upon application of the indicated taste stimuli to the legs of the flies (n=8 for both “5b” and “5s” tarsal neurons). For the statistical analysis the response data for each stimulus are compared; *ns*: non-significant, $^{**}P<0.01$, $^{***}P<0.001$ (Wilcoxon rank sum test).

(d) Fraction of w^{1118} flies (n=68) showing the proboscis extension reflex (PER) to the tastants indicated when applied to the legs (100 mM $NaHCO_3$ at pH 6.5 or pH 8.5, 100 mM sucrose). Error bars represent the ±95% binomial confidence intervals; $^{*}P<0.05$, $^{***}P<0.001$ (Fisher exact test).

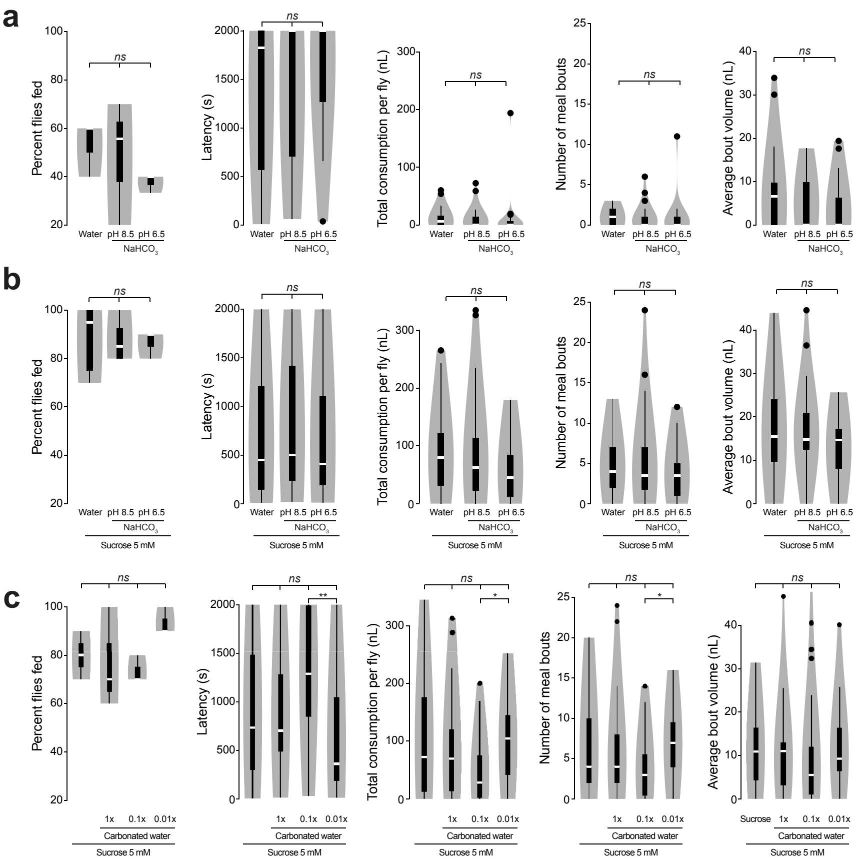

Supplementary Figure 5. Analysis of the influence of carbonation on feeding by Expresso.

(a) Feeding success, latency to first bout, total consumption per fly, number of meal bouts and average bout volume for male w^{1118} flies (n=30 per tastant) feeding from water, 100 mM $NaHCO_3$ pH 8.5 or 100 mM $NaHCO_3$ pH 6.5 solutions. Pairwise comparisons using Tukey and Kramer (Nemenyi) test with Tukey-Dist approximation for independent samples; *ns*: non-significant.

(b) Feeding success, latency to first bout, total consumption per fly, number of meal bouts and average bout volume for male w^{1118} flies (n=30 per tastant) feeding from solutions containing 5 mM sucrose, 5 mM sucrose + 100 mM $NaHCO_3$ pH 8.5 or 5 mM sucrose + 100 mM $NaHCO_3$ pH 6.5 solutions. Pairwise comparisons using Tukey and Kramer (Nemenyi) test with Tukey-Dist approximation for independent samples.

(c) Feeding success, latency to first bout, total consumption per fly, number of meal bouts and average bout volume for male w^{1118} flies (n=30 per tastant) feeding from solutions containing 5 mM sucrose or 5 mM sucrose + the indicated dilutions (v/v) of commercial carbonated water (Supplementary Table 2). Pairwise comparisons using Tukey and Kramer (Nemenyi) test with Tukey-Dist approximation for independent samples: *P<0.05, **P<0.01.

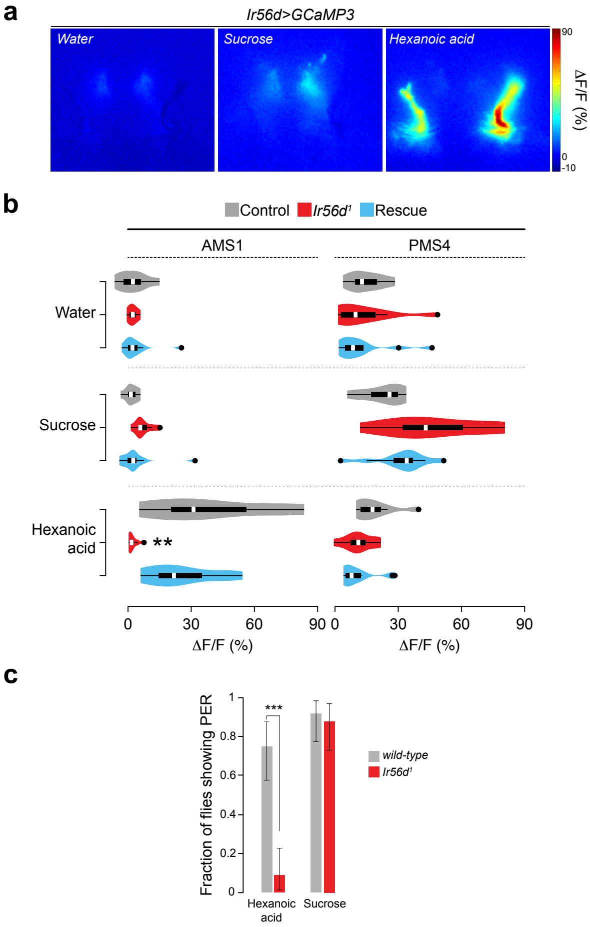

Supplementary Figure 6. IR56d is required for physiological and behavioural responses to hexanoic acid.

(a) Colour-coded images (reflecting the maximal GCaMP3 fluorescence intensity changes) in *w;Bl/+;UAS-GCaMP3,Ir56d-Gal4/+* animals stimulated with distilled water, 1 M sucrose and 1% (v/v) hexanoic acid.

(b) Quantification of changes in GCaMP3 fluorescence (ΔF/F) upon stimulation with the indicated chemicals (as in (a)) to the proboscis of the flies. Genotypes: Control: *w;Bl/+;UAS-GCaMP3,Ir56d-Gal4/+* (n=7); Mutant: *w;Ir56d^{1}/Ir56d^{1};UAS-GCaMP3,Ir56d-Gal4/+* (n=8); Rescue: *w;Ir56d^{1},UAS-Ir56d/Ir56d^{1};UAS-GCaMP3,Ir56d-Gal4/+* (n=11). For the statistical analysis the response data for each stimulus are compared with water; only significant differences are shown: **P<0.01 (Wilcoxon rank sum test with Bonferroni correction for multiple comparisons).

(c) Fraction of *w^{1118}* (n=36) or *Ir56d^{1}* mutant (n=33) flies showing proboscis extension reflex (PER) to 1% (v/v) hexanoic acid and 100 mM sucrose. Error bars represent the ±95% binomial confidence intervals; ***P<0.001 (Fisher exact test).

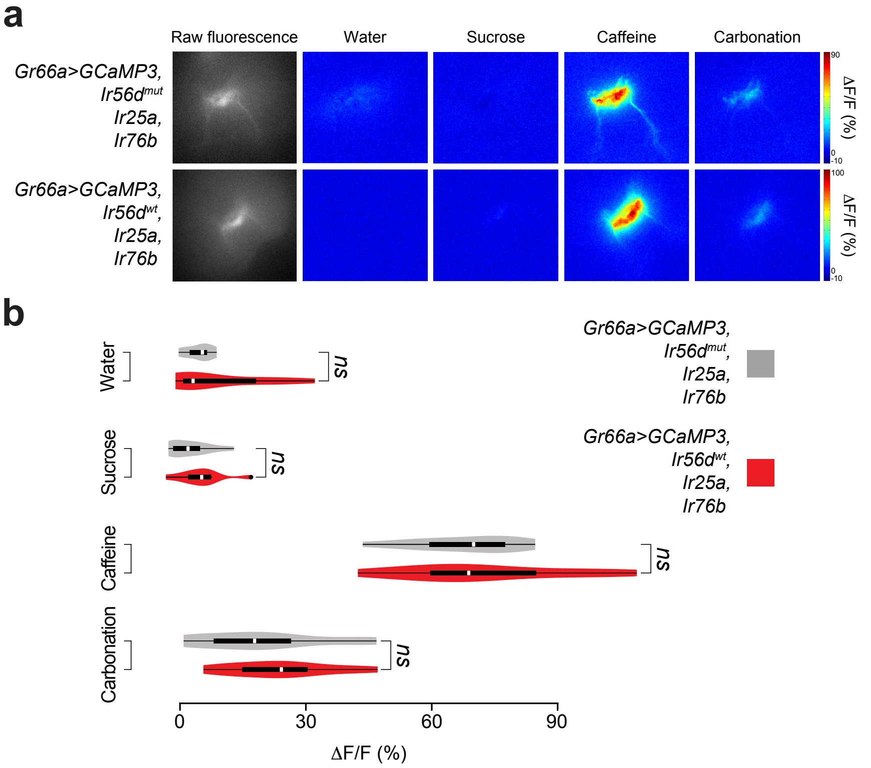

Supplementary Figure 7. Expression of IR56d, IR25a and IR76b in bitter-sensing neurons is not sufficient to confer carbonation sensitivity.

(a) Colour-coded images (reflecting the maximal GCaMP3 fluorescence intensity changes; scale bar on the far-right) in *w;UAS-Ir25a,Gr66a-Gal4/UAS-Ir56d^{mut},UAS-Ir76b;UAS-GCaMP3/+* and *w;UAS-Ir25a,Gr66a-Gal4/UAS-Ir56d^{wt},UAS-Ir76b;UAS-GCaMP3/+* animals stimulated with distilled water, 1 M sucrose, 100 mM caffeine and a carbonated solution. The *UAS-Ir56d^{mut}* transgene contains a frameshift mutation and is predicted to encode a truncated, non-functional receptor; *UAS-Ir56d^{wt}* is the same transgene used in the rescue experiments (Fig. 6c).

(b) Quantification of changes in GCaMP3 fluorescence (ΔF/F) upon stimulation with the indicated chemicals (as in (a)) to the proboscis of flies of the genotypes: *w;UAS-Ir25a,Gr66a-Gal4/UAS-Ir56d^{mut},UAS-Ir76b;UAS-GCaMP3/+* (n=10) and *w;UAS-Ir25a,Gr66a-Gal4/UAS-Ir56d^{wt},UAS-Ir76b;UAS-GCaMP3/+* (n=9). For the statistical analysis, response data for each pair are compared; *ns*: non-significant (Wilcoxon rank sum test with Bonferroni correction for multiple comparisons).

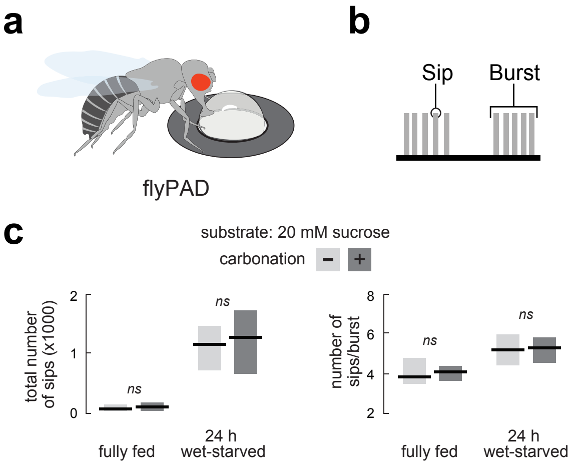

Supplementary Figure 8. Analysis of the influence of carbonation on feeding by flyPAD.
(a) Schematic of the flyPAD assay.
(b) Schematic of the microstructure of feeding behaviour that can be detected with flyPAD. Sips (representing a contact of the proboscis with food) are grouped into feeding bursts.
(c) Total number of sips and number of sips per burst of w^{1118} flies (n=26-60), from 20 mM sucrose solution without (-) or with (+) commercial carbonated water. Boxes represent median with upper/lower quartiles; pairs were compared using Wilcoxon rank-sum test; *ns*: non-significant.
